# Identification of Quantitative Trait Loci Hotspots Affecting Agronomic Traits and High-Throughput Vegetation Indices in Rainfed Wheat

**DOI:** 10.3389/fpls.2021.735192

**Published:** 2021-09-20

**Authors:** Rubén Rufo, Andrea López, Marta S. Lopes, Joaquim Bellvert, Jose M. Soriano

**Affiliations:** ^1^Sustainable Field Crops Programme, Institute for Food and Agricultural Research and Technology (IRTA), Lleida, Spain; ^2^Efficient Use of Water in Agriculture Program, Institute for Food and Agricultural Research and Technology (IRTA), Parc Científici TecnològicAgroalimentari de Gardeny (PCiTAL), Fruitcentre, Lleida, Spain

**Keywords:** wheat, yield components, vegetation indices, marker trait association, candidate genes

## Abstract

Understanding the genetic basis of agronomic traits is essential for wheat breeding programs to develop new cultivars with enhanced grain yield under climate change conditions. The use of high-throughput phenotyping (HTP) technologies for the assessment of agronomic performance through drought-adaptive traits opens new possibilities in plant breeding. HTP together with a genome-wide association study (GWAS) mapping approach can be a useful method to dissect the genetic control of complex traits in wheat to enhance grain yield under drought stress. This study aimed to identify molecular markers associated with agronomic and remotely sensed vegetation index (VI)-related traits under rainfed conditions in bread wheat and to use an *in silico* candidate gene (CG) approach to search for upregulated CGs under abiotic stress. The plant material consisted of 170 landraces and 184 modern cultivars from the Mediterranean basin. The collection was phenotyped for agronomic and VI traits derived from multispectral images over 3 and 2 years, respectively. The GWAS identified 2,579 marker-trait associations (MTAs). The quantitative trait loci (QTL) overview index statistic detected 11 QTL hotspots involving more than one trait in at least 2 years. A CG analysis detected 12 CGs upregulated under abiotic stress in six QTL hotspots and 46 downregulated CGs in 10 QTL hotspots. The current study highlights the utility of VI to identify chromosome regions that contribute to yield and drought tolerance under rainfed Mediterranean conditions.

## Introduction

Wheat (*Triticum aestivum* L.) is the most common cultivated crop worldwide. It is grown on 216 million hectares of land, and its global production of 765 million tons of grain provides 19% of calories and 21% of the protein in the human diet (Faostat 2019, http://www.fao.org/faostat). To cover the expected food demand of a world population that will increase up to 60% by 2050, wheat production needs to be increased by 1.7% per year (Leegood et al., 2010). Achieving this objective will not be easy, considering the expected negative effects of climate change on wheat yield, particularly in areas, such as the Mediterranean basin, where a rise in temperature by 3–5°C and a decrease in the annual rainfall by 25–30% have been predicted (Giorgi and Lionello, 2008). An increasing frequency and severity of terminal drought stress will reduce grain weight, grain quality, and wheat yield (Araus et al., 2002; Slafer et al., 2005; Kulkarni et al., 2017). Therefore, there is a need to improve the identification of genotypes that are able to maintain acceptable levels of yield and yield stability in semiarid environments, which have been identified as the most sensitive to the effects of climate change (Rufo et al., 2021). The release of improved cultivars with enhanced drought adaptation will be critical for breeding programs focusing on wheat adaptability and stability under rainfed conditions (Graziani et al., 2014; Bhatta et al., 2018).

The recent progress in high-throughput phenotyping (HTP) based on the use of multispectral images acquired from unmanned aerial vehicles (UAVs) has increasingly improved the assessment of agronomic traits (Gracia-Romero et al., 2017; Xie and Yang, 2020; Gomez-Candon et al., 2021; Rufo et al., 2021) on large germplasm collections in a rapid, cost-effective, and high spatial resolution way (Duan et al., 2017), as it allows for the estimation of various plant traits using non-intrusive and non-destructive technology (White et al., 2012; Rufo et al., 2021). Remote sensing has attracted growing interest in breeding programs since it can deliver detailed information about biophysical crop traits in many situations to cope with the current phenotyping bottleneck (Araus and Cairns, 2014; Juliana et al., 2019; Bellvert et al., 2021). Some studies have demonstrated the use of vegetation indices (VI) to indirectly detect wheat plants under water stress due to a decrease in vegetative growth (Condorelli et al., 2018). Others have demonstrated the use of energy balance models to estimate the actual water status (Gomez-Candon et al., 2021). When VIs are derived from multispectral cameras, they are obtained from the combination of wavelengths located at the visible, red-edge and near-infrared (NIR) regions of the light spectrum (Kyratzis et al., 2017). These wavelengths allow for discerning differences in vegetative greenness, rate of senescence, photosynthetic efficiency, and stay-green duration (Stenberg et al., 2004; Babar et al., 2006; Lopes and Reynolds, 2012). It has been stated that anthesis (A) and milk grain are the most suitable growth stages for the assessment of agronomic traits on a plot-by-plot basis (Aparicio et al., 2002; Royo et al., 2003). The use of HTP as a suitable and accurate predictor of agronomic traits, such as phenology, grain filling duration, biomass, and yield will provide unique opportunities to increase the power of quantitative trait loci (QTL) discovery by increasing the number of genotypes included in the analysis (Juliana et al., 2019). This method will increase the frequency of rare alleles of potential interest to improve wheat adaptation to various environmental conditions.

The dissection of the genetic and molecular basis of complex traits, such as yield and drought stress tolerance through complementary approaches, such as QTL mapping and genome-wide association studies (GWAS) or association mapping (AM) is essential in breeding programs. GWAS is based on linkage disequilibrium (LD; Flint-Garcia et al., 2003), and it is a powerful approach that provides high mapping resolution due to the higher recombination events analyzed in comparison with biparental mapping (Soriano et al., 2017; Qaseem et al., 2019). The AM has been used to identify genomic regions related to drought and heat tolerance in durum and bread wheat (Maccaferri et al., 2016; Valluru et al., 2017). Several studies have been conducted to investigate the genetic basis of grain yield and yield-related traits in bread wheat under water-stress conditions using AM (Edae et al., 2014; Gizaw et al., 2018; Qaseem et al., 2019; Mérida-García et al., 2020). The release of genome sequences for emmer wheat (Avni et al., 2017), bread wheat (IWGSC, 2018), and durum wheat (Maccaferri et al., 2019) and the availability of open databases of RNA sequencing (RNA-seq) experiments (Ramírez-González et al., 2018) have made it possible to use a candidate gene (CG) approach to find targets within QTL intervals without performing new functional studies.

The aim of the current study is to identify molecular markers linked to important agronomic traits, vegetation indices (VIs), and plant features related to drought resistance assessed by HTP, to define the most important QTL hotspots for such traits and to perform *in silico* detection of the underlying CG in those genomic regions.

## Materials and Methods

### Plant Material and Field Trials

A germplasm collection of 354 bread wheat (*Triticum aestivum* L.) genotypes from the MED6WHEAT IRTA panel described by Rufo et al. (2019) was used in this study, of which 170 corresponded to landraces and 184 to modern varieties collected and adapted to 24 and 19 Mediterranean countries, respectively (Supplementary Table 1). The panel is structured into six genetic subpopulations (SPs) and 38 genotypes that remained admixed (Rufo et al., 2019). SP1: west Mediterranean landraces (43 accessions); SP2: north Mediterranean landraces (59 accessions); SP3: east Mediterranean landraces (42 accessions); SP4: France–Italy modern germplasm (82 accessions); SP5: Balkan modern varieties (24 accessions); and SP6: CIMMYT-ICARDA-derived varieties (62 accessions).

The field trials were conducted at Gimenells, Lleida (41°38' N and 0°22' E, 260 m.a.s.l), northeastern Spain, under rainfed conditions for three consecutive seasons: 2016, 2017, and 2018. Average minimum and maximum monthly temperatures and rainfall were calculated from daily data recorded for a weather station close to the experimental fields. Climatic data (rainfall and temperature) for the last 15 years corresponding to the weather station in Gimenells, Lleida (Spain) were downloaded from https://ruralcat.gencat.cat/web/guest/agrometeo.estacions. Experiments followed a non-replicated augmented design with two replicated checks (*cv*. “Anza” and “Soissons”) at a ratio of 1:4 between checks and tested genotypes and in 3.6 m^2^ plots with eight rows spaced 0.15 m apart. The sowing density was adjusted to 250 germinable seeds m^−2^, and the sowing dates were December 2, 2015; November 21, 2016; and November 15, 2017, whereas harvesting dates were July 7, 2016; July 5, 2017; and July 5, 2018. Weeds and diseases were controlled following standard practices at the site.

### Agronomic Data

The following traits were measured across the 3 years (2016, 2017, and 2018) according to the protocol described by Rufo et al. (2021): grain yield (GY, t ha^−1^), number of spikes per m^2^ (NSm^2^), number of grains per m^2^ (NGm^2^), thousand kernel weight (TKW, g), aboveground biomass at physiological maturity (biomass, t DM ha^−1^), harvest index (HI), plant height (PH, cm) and early vigor (estimated as green area, GA). The HI was calculated as the ratio between grain and plant weights in a 1-m long row sample. The PH was measured at maturity for three main stems per plot and was measured from the soil to the top of the spike, excluding the awns. Early vigor was calculated by integrating the green area (GA) values obtained by ground-based red, green, and blue (RGB) images taken every 14 days as described by Casadesús and Villegas (2014) from emergence until the detection of the first node. Finally, days from sowing to anthesis (A) (DSA, GS65) and grain filling duration (GFD, GS87) were measured on each plot based on the growth stage (GS) scale of Zadoks et al. (1974). The GSs were achieved when at least 50% of the plants in each plot reached the stage.

### Image Acquisition

Image acquisition was conducted with a multispectral camera (Parrot Sequoia) (Parrot, Paris, France) installed onboard an UAV (DJI S800 EVO hexacopter, Nanshan, China). Images were acquired during 2 years, on April 21, 2017 and May 19, 2017 and on April 17, 2018 and May 18, 2018. Flights were always conducted at ~12:00 solar time under sunny conditions and with a wind speed below 12 m/s. The UAV flew at a height of 40 m above ground level (agl) and with a flight plan of 80/60 frontal and side overlap. The multispectral camera has four spectral bands located at wavelengths of 550 ± 40 nm (green), 660 ± 40 nm (red), 735 ± 10 nm (red edge), and 790 ± 40 (near infrared). The camera yields a resolution of 1,280 × 960 pixels. All images were radiometrically corrected through an external incident light sensor that measured the irradiance levels of light at the same bands as the sensor, as well as with *in situ* spectral measurements in ground calibration targets (black, white, soil, and grass). Spectral measurements were conducted with a Jaz spectrometer (Ocean Optics, Inc., Dunedin, FL, USA). Jaz has a wavelength response from 200 to 1,100 nm and an optical resolution of 0.3–10.0 nm. The calibration of the spectrometer measurements was taken using a reference panel (white color Spectralon™). Geometrical correction was conducted by using ground control points (GCPs) and measuring the position in each with a handheld global positioning system (GPS) (Geo7x, Trimble GeoExplorer series, Sunnyvale, CA, USA). All images were mosaicked using AgisoftPhotoscan Professional version 1.6.2 (Agisoft LLC., St. Petersburg, Russia) software and geometrically and radiometrically corrected with QGIS 3.2.0 (USA, http://www.qgis.org). Then, six spectral vegetation indices (VIs) were carefully selected based on their significance in relation to certain plant physiology features in wheat (Table 1). In addition, the leaf area index (LAI) was measured using a portable ceptometer (AccuPAR model LP-80, decagon devices Inc., Pullman, WA, USA) from 13:00 to 15:00 (local time) on each image acquisition date in 64 different plots of each set of landrace and modern set. Then, the LAI was estimated for each plot in the whole collection through the MTVI2, following the methodology described by Rufo et al. (2021) and Gomez-Candon et al. (2021). All VIs were assessed in 2017 and 2018 through UAV multispectral images at two growth stages: (1) when all the plots reached anthesis (A) (VI___A) and (2) postanthesis (PA) at the milk and dough developmental stages (VI_PA).

**Table 1:** Spectral vegetation indices assessed in this study.

**Vegetation index**	**Band center (nm)**	**Spectral band**	**References**
**Structural indices**			
Normalized Difference Vegetation Index (NDVI)	790, 660	NIR, Red	Rouse et al., 1974
Renormalized Difference Vegetation Index (RDVI)	790, 660	NIR, Red	Roujean and Breon, 1995
Improved Soil Adjusted Vegetation Index (MSAVI)	790, 660	NIR, Red	Qi et al., 1994
Modified Triangular Vegetation Index (MTVI2)	790, 660, 550	NIR, Red, Green	Haboudane et al., 2004
Green Normalized Difference Vegetation Index (GNDVI)	790, 550	NIR, Green	Gitelson et al., 1996
**Chlorophyll indices**			
Transformed CARI/Optimized Soil adjusted Vegetation Index (TCARI/OSAVI)	790, 735, 660, 550	NIR, Red Edge, Red, Green	Haboudane et al., 2002

### Genotyping

The panel was genotyped with 13,177 SNP markers using the Illumina Infinium 15K Wheat SNP Array at Trait Genetics GmbH (Gatersleben, Germany), and 11,196 markers were ordered according to the SNP map developed by Wang et al. (2014). To reduce the risk of errors in further analyses, markers and accessions were analyzed for the presence of duplicated patterns and missing values. After excluding markers with more than 25% missing values and with a minor allele frequency (MAF) lower than 5%, a total of 10,090 SNPs were used for mapping purposes.

### Statistical Analyses

Phenotypic data were fitted to a linear mixed model considering the check cultivars as the fixed effect, and the row and column number and accessions as random in the model for each environment following the MIXED procedure of the SAS-STAT statistical package (SAS Institute Inc., Cary, NC, USA).


$$\begin{array}{l} y=x\beta +z\gamma +\varepsilon \; \end{array}$$

where β is an unknown vector of fixed-effects parameters with known design matrix x, γ is an unknown vector of random-effects parameters with known design matrix z, and ε is an unknown random error vector whose elements are no longer required to be independent and homogeneous. Restricted maximum likelihood (REML) was used to estimate the variance components and to produce the best linear unbiased predictors (BLUPs) for agronomic traits and VIs (Supplementary Table 2).

To assess the differences between years and genetic subpopulations, one-way ANOVAs were conducted for the whole collection. The broad sensed heritability (H^2^) was estimated following Knapp et al. (1985).


$$\begin{array}{l} H^{2}=\frac{\sigma_{G}^{2}}{\sigma_{G}^{2}+\sigma_{E}^{2}+\sigma_{GE}^{2}} \end{array}$$

where $\sigma_{G}^{2}$ is the genotypic variance, $\sigma_{E}^{2}$ is the variance due to the environmental (year) effect, and $\sigma_{GE}^{2}$ is the is the variance for the interaction of genotype with environment.

Least squares means were calculated and compared using the Tukey's HSD test. Pearson's correlation coefficients were calculated among the evaluated traits. Mean phenotypic values across the 3 years were used to perform a hierarchical cluster analysis by the Ward method (Ward, 1963). Analyses of variance and mean differences were carried out using the JMP v14.2.0 statistical package (SAS Institute, Inc., Cary, NC, USA), considering a significance level of alpha = 0.05.

### Marker Trait Associations

A GWAS with 10,090 SNP markers was conducted on the whole germplasm collection using Tassel 5.0 software (Bradbury et al., 2007) for all agronomic and VI traits per year and across the three growing seasons. A mixed linear model (MLM) was fitted using a principal component analysis (PCA) matrix with six principal components as the fixed effect and a kinship (k) matrix as the random effect (PCA + K model) at the optimum compression level based on the groups defined by the kinship matrix. Compression levels range from “no compression” (compression = 1) when each genotype belongs to its own group, to “maximum compression” (compression = *n*) when all genotypes belong to the same group. In addition, the (A) date was incorporated as a cofactor in the analysis, as reported in previous studies (Crowell et al., 2016; Condorelli et al., 2018; Soriano et al., 2021). Manhattan plots were generated using the R script, CMplot (https://github.com/YinLiLin/CMplot). A false discovery rate (FDR) threshold (Benjamini and Hochberg, 1995) was established at –log_10_ p > 4.8 (*p* < 0.05), using 3,696 markers according to the results of the linkage disequilibrium (LD) decay (Rufo et al., 2019). Besides, a frequently used threshold was established at –log_10_ *P* > 3, as previously reported in the literature (Wang et al., 2014, 2020; Condorelli et al., 2018; Mangini et al., 2018; Sukumaran et al., 2018). Confidence intervals (CIs) for MTAs were estimated for each chromosome according to the LD decay reported by Rufo et al. (2019) using the formula reported by Chardon et al. (2004).


$$\begin{array}{l} S_{i}^{2}={\left(\frac{CI}{3.92}\right)}^{2} \end{array}$$

where CI corresponded with the LD decay for each chromosome. To simplify the MTA information, the associations were grouped into QTL hotspots. To define a hotspot, the density of MTAs along the chromosome was calculated as the QTL overview index (Chardon et al., 2004) for each cM of the genetic map reported by Wang et al. (2014).


$$\begin{array}{l} U=\frac{\frac{nbQTL}{nbE}}{Total\;length\;of\;map} \end{array}$$

where nbQTL is the number of QTLs and nbE is the total number of experiments.

### Gene Annotation and *in silico* Gene Expression Analysis

Gene annotation for the target region of QTL hotspots was performed using the gene models for high-confidence genes reported for the wheat genome sequence (IWGSC, 2018), available at https://wheat-urgi.versailles.inra.fr/Seq-Repository/Annotations. Physical distances were estimated using the genetic distances from the markers flanking the CIs of each QTL hotspot.

*In silico* expression analysis and the identification of upregulated gene models were carried out using the RNA-seq data available at http://www.wheat-expression.com/ (Ramírez-González et al., 2018) for the following studies: (1) drought and heat stress time-course in seedlings, (2) spikes with water stress, and (3) seedlings with polyethylene glycol (PEG) to simulate drought.

Gene Ontology (GO) data were retrieved from the high-confidence gene annotation at https://wheat-urgi.versailles.inra.fr/Seq-Repository/Annotations.

## Results

### Environmental Conditions

**Figure 1:**
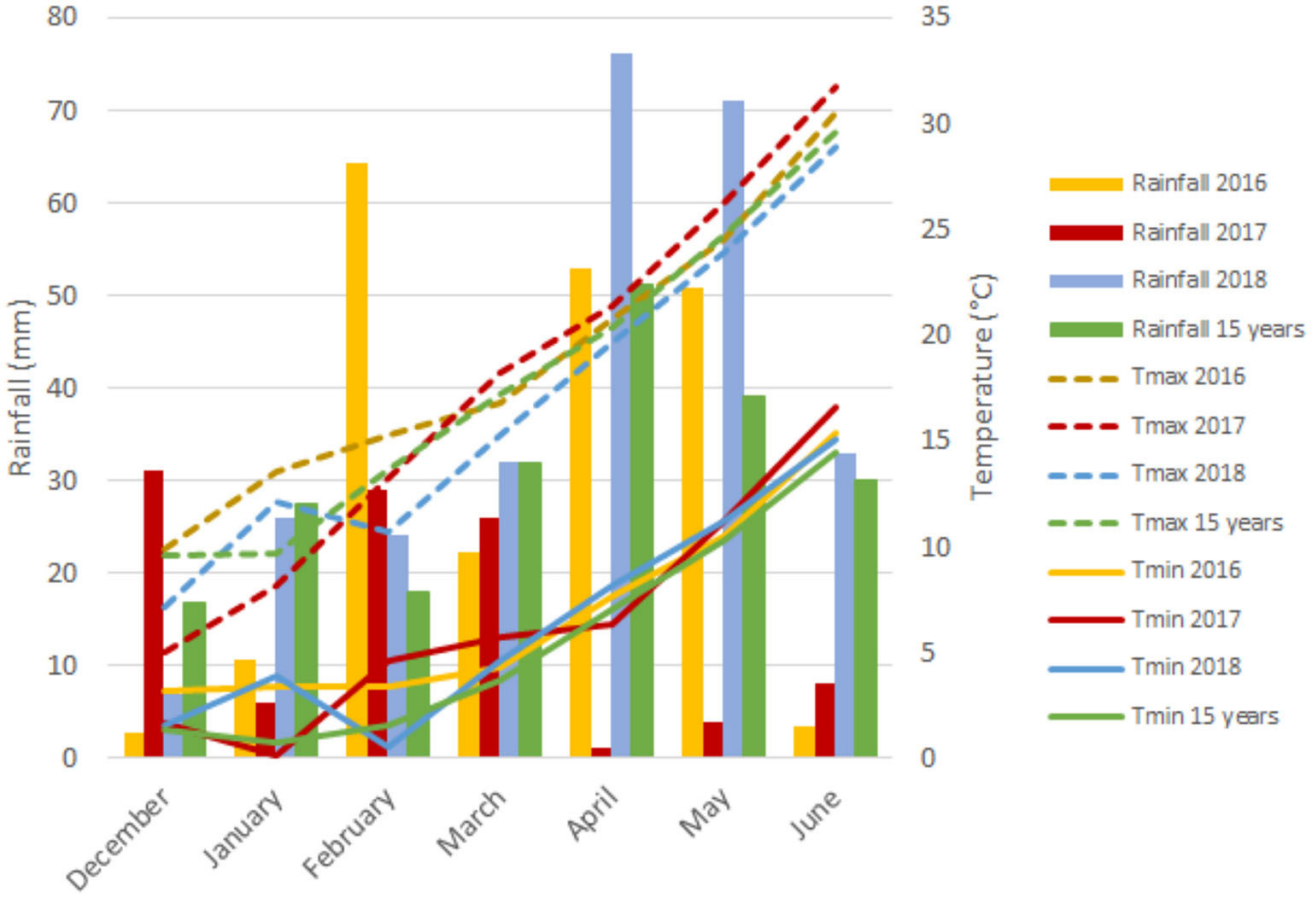
Monthly rainfall (mm) and minimum (Tmin) and maximum (Tmax) temperatures during the growth cycle of each growing season.

The experimental site has a typical Mediterranean climate characterized by an irregular pattern of yearly rainfall distribution, low temperatures in winter that rise sharply in spring, and high temperatures continuing until the end of the crop cycle. Figure 1 represents a graphical summary of the rainfall and maximum and minimum temperatures during the crop cycle across the 3 years of field trials and the average of the last 15 years. Although precipitation values were representative of long-term data from the region for each growing season, the year 2017 was considered exceptionally dry due to the low rainfall received. The year 2018 was characterized as the wettest from December (sowing) to June (physiological maturity) with 269 mm of rainfall, whereas the first and second growing seasons with 207 and 105 mm, respectively, of rainfall were rather dry, suffering severe water scarcity during the grain filling period with only 5 mm of precipitation.

### Phenotypic Analyses

A summary of the genetic variation is shown in Table 2, Supplementary Table 3 for agronomic and vegetation indices (VIs)-related traits. Agronomic traits showed coefficients of variation (CV) ranging from 36.6% for grain yield to 6.3% for days to anthesis (A). VIs showed higher CVs during postanthesis (PA) with values at A ranging from 17.4% for leaf area index (LAI) to 2.1% for normalized difference vegetation index (NDVI), and PA ranging from 54.0% for LAI to 15.3% for green normalized difference vegetation index (GNDVI). Agronomic traits showed higher values for heritability than VI for most of the traits. For the agronomic traits, heritability ranged from 0.9 for yield to 0.1 for green area (GA), whereas for VIs, heritability ranged between 0.41 and 0.05 for TCARI/OSAVI and RDVI, respectively at A, and between 0.46 and 0.03 for TCARI/OSAVI and MTVI, 2 respectively during PA.

**Table 2:** Summary statistics of the agronomic traits, leaf area index (LAI), and VIs.

	**Yield**	**HI**	**Biomass**	**NSm^**2**^**	**NGm^**2**^**	**TKW**	**PH**	**GA**	**GS65**	**GFD**
Min	0.5	0.1	4.2	200	1,122	12.7	70.2	16.2	132	18
Max	14.4	0.9	33.9	973.3	51,548	58.8	163.7	56.6	180	71
Mean	7.7	0.4	17.2	541.9	20,199	38.7	105.2	35.8	158.2	34
SD	2.8	0.1	3.9	115.4	7,087	6.6	18	9	10	5.3
CV (%)	36.6	22.2	22.7	21.3	35.1	17.1	17.1	25	6.3	15.7
h^2^	0.9	0.8	0.4	0.5	0.8	0.6	0.8	0.1	0.3	0.5
	**LAI**	**NDVI**	**RDVI**	**MSAVI**	**MTVI2**	**TCARI/OSAVI**	**GNDVI**			
**Anthesis**										
Min	0.1	0.8	0.55	0.61	0.67	−7.26	0.77			
Max	7.67	1	0.87	0.95	1	0.22	0.94			
Mean	5.53	0.94	0.69	0.8	0.88	−0.23	0.87			
SD	0.96	0.02	0.09	0.11	0.12	0.02	0.03			
CV (%)	17.4	2.1	13	13.7	13.6	8.7	3.4			
h^2^	0.18	0.4	0.05	0.05	0.13	0.41	0.13			
**Postanthesis**										
Min	0.72	0.48	0.29	0.28	0.24	−3.89	0.5			
Max	6.62	0.95	0.76	0.86	0.86	0.44	0.92			
Mean	3.26	0.77	0.54	0.61	0.6	−0.03	0.72			
SD	1.76	0.13	0.13	0.18	0.21	0.01	0.11			
CV (%)	54	16.9	24.1	29.5	35	40	15.3			
h^2^	0.04	0.08	0.06	0.04	0.03	0.46	0.09			

*SD, standard deviation; CV, coefficient of variability; h^2^, heritability; GS65, number of days from sowing to anthesis (A); GFD, grain filling duration; HI, harvest index; NSm^2^, number of spikes per square meter; NGm^2^, number of grains per square meter; TKW, thousand kernel weight; PH, plant height; GA, green area*.

The results of ANOVAs for the agronomic traits measured during the three growing seasons are shown in Table 3. The percentage of variability explained by year was the highest for GA (81.6%) and GS65 (67.9%), while the sum of squares of subpopulation (SP) was the highest for yield (76.5%), NGm^2^ (65.0%), harvest index (HI) (62.6%), plant height (PH) (61.4%), and GS87 (59.0%). Finally, the highest percentage explained by the interaction between year and SP was found fo rbiomass, NSm^2^ and thousand kernel weight (TKW), reaching 86.2, 84.9, and 71.0%, respectively. Significant differences were found between SPs for all traits. The year and the year × SP interactions were also significant for all traits, except for HI.

**Table 3:** Analysis of variance for grain yield, harvest index (HI), biomass, number of spikes per square meter (NSm^2^), number of grains per square meter (NGm^2^), thousand kernel weight (TKW), plant height (PH), green area (GA), number of days from sowing to anthesis (A) (GS65), and grain filling duration (GFD, GS87) for the three years of field trials.

**SS (%)**	**Yield**	**HI**	**Biomass**	**NSm^**2**^**	**NGm^**2**^**	**TKW**	**PH**	**GA**	**GS65**	**GFD**
Year	1.3^**^	0.1	5.2^**^	8.0^**^	6.5^**^	10.5^**^	2.6^**^	81.6^**^	67.9^**^	8.7^**^
SP	76.5^**^	62.6^**^	8.6^**^	7.1^**^	65.0^**^	18.5^**^	61.4^**^	2.2^**^	30.4^**^	59.0^**^
Year × SP	22.2^**^	37.3	86.2^**^	84.9^*^	28.5^**^	71.0^*^	36.0^**^	16.2^**^	1.7^**^	32.3^**^

*SS, sum of squares; SP, subpopulation*.

* *p < 0.01;*

** *p < 0.001*.

Table 4 shows the results of the ANOVA for the VIs and LAI estimated through the MTVI2 at the A and PA stages during 2017 and 2018. Differences between SPs and between years, as well as the year × SP interaction, were statistically significant for all traits in both years. The sum of squares of the year accounted for 1.3% (NDVI) to 92.9% (RDVI) of the variation at A, whereas at PA, the percentages ranged from 10.0% (TCARI/OSAVI) to 92.3% (LAI). The percentages of the total variation explained by SP ranged from 2.3% (RDVI) to 11.0% (TCARI/OSAVI) at A, while they ranged from 1.0% (MTVI2) to 11.2% (TCARI/OSAVI) PA. Year was the most important criterion for explaining the variations in LAI, RDVI, MSAVI, MTVI2, and GNDVI in the two growth stages. SP explained the least percent of variation at both growth stages for all traits. The year x SP interaction accounted for 4.8% (RDVI) to 89.9% (NDVI) of the model variance at A, with the highest values for NDVI and TCARI/OSAVI. The variance explained by the year × SP interaction at PA ranged from 6.6% (LAI) to 78.8% (TCARI/OSAVI).

**Table 4:** Analyses of variance for the LAI estimated through MTVI2 and all the VIs calculated at the anthesis (A) and postanthesis (PA) stages in 2017 and 2018.

**SS (%)**	**LAI**	**NDVI**	**RDVI**	**MSAVI**	**MTVI2**	**TCARI/OSAVI**	**GNDVI**
**Anthesis**							
Year	62.6^**^	1.3^*^	92.9^**^	88.6^**^	71.1^**^	15.6^**^	67.7^**^
SP	8.9^**^	8.8^**^	2.3^**^	2.7^**^	8.9^**^	11.0^**^	8.0^**^
Year × SP	28.5^**^	89.9^**^	4.8^**^	8.7^**^	20.0^**^	73.4^**^	24.3^**^
**Postanthesis**							
Year	92.3^**^	85.3^**^	88.2^**^	90.8^**^	91.8^**^	10.0^**^	83.4^**^
SP	1.1^**^	2.9^**^	2.2^**^	1.4^**^	1.0^**^	11.2^**^	4.8^**^
Year × SP	6.6^**^	11.8^**^	9.6^**^	7.8^**^	7.2^**^	78.8^**^	11.8^**^

*SS, % of the sum of squares; SP, subpopulation*.

* *p < 0.01;*

** *p < 0.001*.

The mean values of phenotypic traits for each year and SP are shown in Table 5. Yearly means showed that the highest yield was in 2016, a year in which the yield components NSm^2^, NGm^2^, and TKW reached intermediate values between those obtained in the two subsequent years. The shortest duration of the preanthesis period and the longest grain filling duration (GFD) were also observed in 2016. On the other hand, the lowest yield, NSm^2^ and NGm^2^, the heaviest grains and the shortest GFD were observed in 2017. The GA reached the highest value in 2016, which was characterized as the wettest year during the period from January–March, i.e., the stem elongation stage, when the trait was measured. In contrast, 2017 was the driest year in the same period, which showed the lowest value for GA. In 2017, PH showed maximum values but biomass showed the lowest values at maturity. Finally, in 2018, biomass, the number of spikes, and grains per unit area showed high values, and the cycle until A was the longest.

**Table 5:** Mean values of grain yield, harvest index (HI), biomass, number of spikes per unit area (NSm^2^), number of grains per unit area (NGm^2^), thousand kernel weight (TKW), plant height (PH), green area (GA), number of days from sowing to anthesis (A) (GS65), and grain filling duration (GFD, GS87) in a set of 170 landraces and 184 modern cultivars of bread wheat for each growing season and genetic subpopulation.

	**Yield (t/ha)**	**HI**	**Biomass (t/ha)**	**NSm^**2**^**	**NGm^**2**^**	**TKW (g)**	**PH (cm)**	**GS65**	**GA**	**GFD**
2016	8.0^a^	0.35^a^	17.5^a^	532^b^	20836^a^	38.6^b^	105.8^b^	150^c^	46.7^a^	36^a^
2017	7.4^c^	0.36^a^	16.1^b^	509^b^	17877^b^	41.3^a^	108.4^a^	155^b^	26.6^c^	32^c^
2018	7.9^b^	0.36^a^	18.1^a^	583^a^	21966^a^	36.2^c^	101.6^c^	169^a^	35.8^b^	34^b^
SP1	5.2^e^	0.29^b^	16.6^c^	556^ab^	14065^de^	38.2^bc^	120.4^a^	159^b^	37.5^a^	33 ^c^
SP2	5.9^d^	0.30^b^	16.7^c^	534^b^	15354^d^	39.2^b^	121.9^a^	163^a^	35.9^bc^	31^c^
SP3	4.4^f^	0.29^b^	14.9^d^	569^ab^	13613^e^	32.6^d^	114.9^b^	159^b^	33.5^d^	33^c^
SP4	10.8^a^	0.44^a^	18.5^a^	568^a^	28045^a^	39.3^b^	87.1^d^	158^b^	35.7^c^	35^b^
SP5	9.2^b^	0.43^a^	18.0^abc^	475^c^	21872^b^	43.2^a^	92.1^cd^	160^b^	37.9^a^	35^b^
SP6	9.7^b^	0.42^a^	18.0^ab^	493^c^	23877^b^	42.0^a^	95.7^c^	152^c^	38.1^a^	37^a^
AD	6.7^c^	0.31^b^	17.1^bc^	550^ab^	18414^c^	36.9^c^	111.4^b^	157^b^	36.8^ab^	35^b^

*Data for each subpopulation represent the mean values across the 3 years. Different letters at each growing season or subpopulation indicate significant differences at p ≤ 0.01 using Tukey’s honest significant difference test*.

*SP1, West Mediterranean landraces; SP2, North Mediterranean landraces; SP3, East Mediterranean landraces; SP$, Modern cultivars from France and Italy; SP5, Modern cultivars from Balkans; SP6, Modern cultivars from CIMMYT and ICARDA; AD, admixed genotypes*.

Significant differences in agronomic traits between SPs highlighted the division of the whole set into landraces and modern cultivars (Table 5). Modern SPs (SP4, SP5, and SP6) showed higher values of grain yield and yield components, HI, and biomass than landrace SPs. The highest value for grain yield was observed for SP4, in agreement with its higher number of spikes and grains per unit area. The SP4 showed the lowest grain weight among modern SPs but was not significantly different from the heaviest grains observed in landraces (SP1 and SP2). As expected, landraces were taller than modern cultivars. The SP3 showed the lowest value for GA. For phenology, SP2 took the longest time to reach the A stage, whereas SP6 took the shortest time. In contrast, the GFD was the shortest for SP2 and the longest for SP6. Modern SPs showed a longer GFD than landraces.

The mean values of the VIs and LAI (estimated by MTVI2) at A and at PA for 2017 and 2018 and the different SPs are shown in Table 6. All traits had higher values at A, except for TCARI/OSAVI. For all traits, differences between years were statistically significant at the two stages. The LAI, RDVI, and MSAVI showed the highest mean values in 2018. The mean values for TCARI/OSAVI were the highest in 2017. The year 2017 showed the highest values of MTVI2 and GNDVI at A, but these VIs and NDVI were minimal at PA the same year. Due to saturation of the reflectance, NDVI became insensitive at high LAI values (LAI > 3) in both years at A. LAI, NDVI, RDVI, MSAVI, and MTVI2 significantly differed between landrace and modern cultivar SPs at A, with higher values being recorded in the landraces. However, no pattern was found for VI traits among SPs PA. SP2 and SP4 had higher mean values for all traits PA, with the exception of TCARI/OSAVI.

**Table 6:** Mean values of the LAI estimated by MTVI2 and all the VIs at anthesis (A) and postanthesis (PA) stages in 2017 and 2018 as well as for each genetic subpopulation and the group of admixed genotypes from a set of 170 landraces and 184 modern bread wheat cultivars.

	**LAI**	**NDVI**	**RDVI**	**MSAVI**	**MTVI2**	**TCARI/OSAVI**	**GNDVI**
**Anthesis**							
2017	4.77^b^	0.95^a^	0.59^b^	0.70^b^	0.78^a^	0.03^a^	0.9^a^
2018	6.29^a^	0.94^a^	0.78^a^	0.91^a^	0.98^b^	−0.51^b^	0.84^b^
SP1	5.88^a^	0.96^a^	0.71^a^	0.83^a^	0.92^a^	−0.08^a^	0.87^b^
SP2	5.86^a^	0.96^a^	0.71^a^	0.83^ab^	0.92^ab^	−0.08^a^	0.87^b^
SP3	5.75^a^	0.95^ab^	0.69^b^	0.81^c^	0.91^ab^	−0.02^a^	0.87^b^
SP4	5.27^b^	0.94^bc^	0.67^c^	0.78^d^	0.85^c^	−0.63^b^	0.89^a^
SP5	5.24^b^	0.93^c^	0.67^c^	0.78^d^	0.84^c^	−0.21^a^	0.87^bc^
SP6	5.20^b^	0.94^c^	0.67^c^	0.78^d^	0.84^c^	−0.21^a^	0.86^c^
AD	5.70^a^	0.95^a^	0.69^b^	0.81^bc^	0.90^b^	−0.09^a^	0.87^b^
**Postanthesis**							
2017	1.56^b^	0.65^b^	0.42^b^	0.43^b^	0.39^b^	0.07^a^	0.62^b^
2018	4.95^a^	0.89^a^	0.67^a^	0.79^a^	0.8^a^	−0.13^b^	0.82^a^
SP1	3.21^bc^	0.76^b^	0.56^ab^	0.62^ab^	0.61^ab^	0.06^ab^	0.70^cd^
SP2	3.32^b^	0.80^a^	0.57^a^	0.64^a^	0.64^a^	−0.08^c^	0.73^b^
SP3	2.91^d^	0.74^cd^	0.54^c^	0.60^b^	0.59^bc^	0.14^a^	0.68^d^
SP4	3.50^a^	0.79^a^	0.55^bc^	0.62^b^	0.60^b^	−0.20^d^	0.75^a^
SP5	3.36^ab^	0.77^bc^	0.53^c^	0.60^b^	0.59^bc^	−0.01^abc^	0.73^b^
SP6	3.13^c^	0.74^d^	0.51^d^	0.57^c^	0.57^c^	0.03^abc^	0.70^c^
AD	3.20^bc^	0.76^bc^	0.54^c^	0.60^b^	0.59^b^	−0.01^bc^	0.71^c^

*Years followed by different letters indicate significant differences at p ≤ 0.01 using Tukey's honest significant difference test*.

*SP1, West Mediterranean landraces; SP2, North Mediterranean landraces; SP3, East Mediterranean landraces; SP4, Modern cultivars from France and Italy; SP5, Modern cultivars from Balkans; SP6, Modern cultivars from CIMMYT and ICARDA; AD, admixed*.

**Figure 2:**
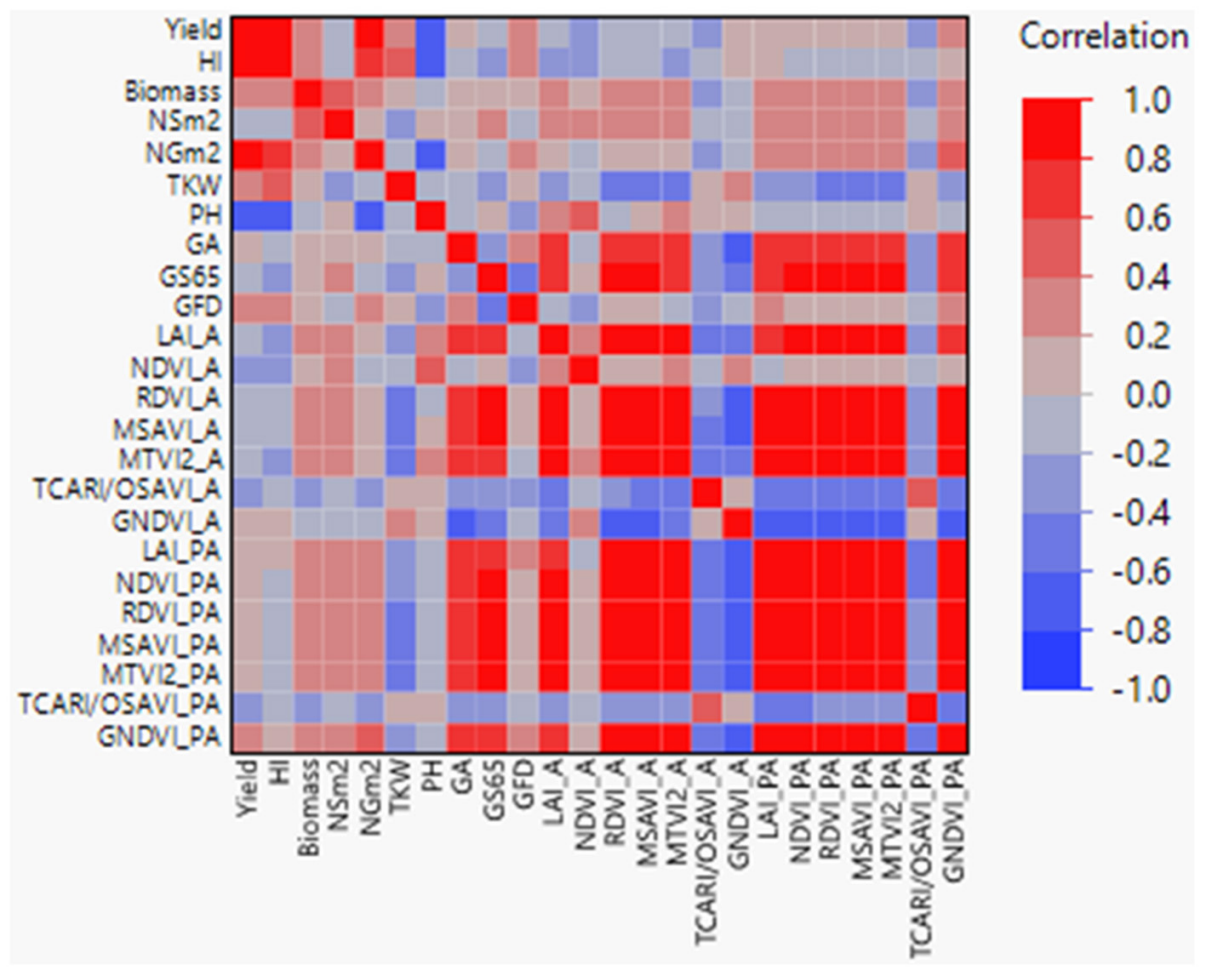
Pearson correlations between agronomic traits, vegetation indices (VIs), and LAI+. GS65, number of days from sowing to A. GFD, grain filling duration; HI, harvest index; NSm2, number of spikes per square meter; NGm2, number of grains per square meter; TKW, thousand kernel weight; LAI, leaf area index; PH, plant height; GA, green area; A, anthesis; PA, postanthesis. Significant correlations at *P* < 0.0001 were established for *r* > 0.45 and *r* < −0.45.

Correlation coefficients between agronomic traits, VIs, and LAI were calculated (Figure 2), showing highly significant coefficients among agronomic traits as yield with NGm^2^ (*r* = 0.90) and PH (*r* = −0.71). Interestingly, when analyzed VIs-related traits against agronomic traits, highly significant coefficients (*r* > 0.61) were found between GS65 and RDVI, MTVI2, GNDVI, LAI, NDVI_PA, and MSAVI_PA, and between GA and MSAVI, GNDVI, LAI, MTVI2, and RDVI_A. In addition, NGm^2^ and PH showed a moderate significant correlation with GNDVI_PA and NDVI_A, respectively (*r* = 0.46).

To quantify the relation between trait variation and population structure, multiple linear regressions were carried out between population structure (*q*_*i*_) coefficients (Rufo et al., 2019) (Table 7) and phenotypic performance for landrace and modern sets separately and both sets were combined. The landrace *R*^2^ values ranged from 0.10 for MSAVI_A to 0.39 for GA, while the modern *R*^2^ values ranged from 0.10 for MTVI2_A to 0.64 for GNDVI_A. When the regressions were conducted on the combined data set, the *R*^2^ values ranged from 0.11 for biomass to 0.60 for NGm^2^. The traits yield and GNDVI_PA showed high *R*^2^ values (>0.35) for each set separately and for the combined set. The highest *R*^2^ values were found in modern set regressions for GNDVI_A, GNDVI_PA, NDVI_PA, and GS65. Among the components of yield, thousand kernel weight (TKW) showed the highest *R*^2^ values in landrace set regressions, while in modern set regressions, NGm^2^ showed the highest *R*^2^ values.

**Table 7:** Relationship between trait variation and population structure (*q*-values) for landrace and modern sets separately and the combined set.

**Trait**	***R*** **^2^ LR vs. MOD**	***R*** **^2^ LR**	***R*** **^2^ MOD**
	***N*** **= 354**	***N*** **= 170**	***N*** **= 184**
GS65	–	–	0.47
GFD	–	–	0.16
Yield	0.59	0.37	0.43
HI	0.47	–	-
Biomass	0.11	–	–
NSm^2^	–	0.12	0.18
NGm^2^	0.60	–	0.42
TKW	–	0.34	0.11
LAI_A	0.20	–	–
NDVI_A	–	–	–
RDVI_A	0.20	–	–
MSAVI_A	0.22	0.10	–
MTVI2_A	0.29	–	0.10
TCARI/OSAVI_A	0.19	–	0.13
GNDVI_A	0.39	–	0.64
LAI_PA	0.20	–	0.39
NDVI_PA	0.14	0.23	0.50
RDVI_PA	–	0.11	0.40
MSAVI_PA	–	0.13	0.44
MTVI2_PA	–	0.11	0.39
TCARI/OSAVI_PA	0.20	0.17	0.17
GNDVI_PA	0.51	0.39	0.60
PH	0.48	–	0.33
GA	–	0.39	–

*GS65, number of days from sowing to anthesis; GFD, grain filling duration; HI, harvest index; NSm^2^, number of spikes per square meter; NGm^2^, number of grains per square meter; TKW, thousand kernel weight; LAI, leaf area index; PH, plant height; GA, green area; A, anthesis; PA, postanthesis; LR, landrace; MOD, modern; N, number of genotypes*.

**Figure 3:**
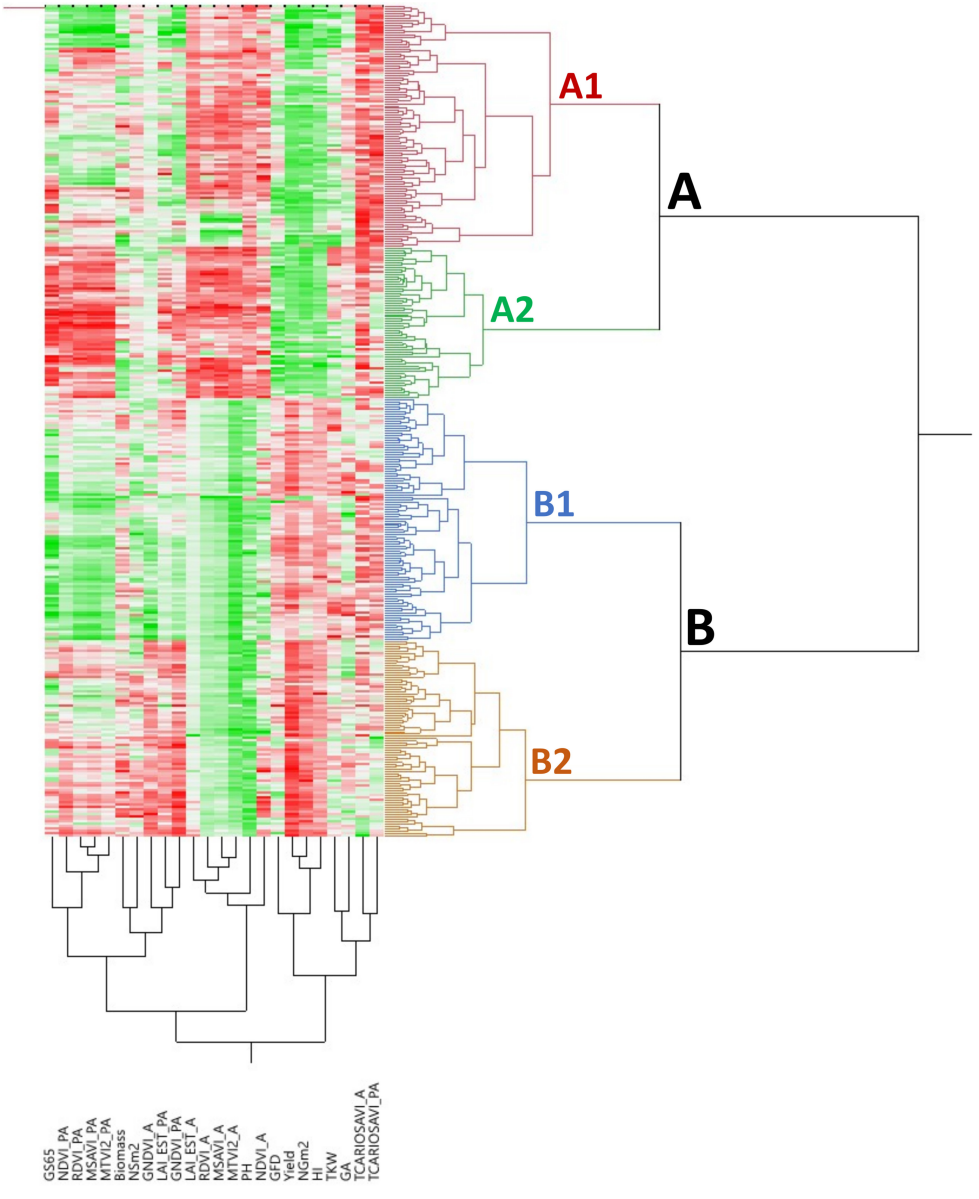
Bidimensional clustering showing the phenotypic relationships between the 354 bread wheat genotypes based on the analyzed traits indicated in the vertical cluster at bottom. Red and green colors in the columns indicate high and low values, respectively. Dark, higher values; light, lower values; white, intermediate values.

The bidimensional clustering shown in Figure 3 represents the relationships among accessions and their mean phenotypic performances (3 years for agronomic traits and 2 years for VIs). The horizontal cluster grouped accessions according to their phenotypic similarity based on the traits in the vertical cluster. Horizontal clustering separated two main clusters: Cluster A was composed only of landraces and Cluster B included modern cultivars and two landraces: *cv* “TRI 11548” from Iraq and *cv* “1170” from Turkey. Cluster A was characterized by lower yield and yield components, except NSm^2^, lower biomass, a shorter GFD but longer GS65, and taller plants than Cluster B, but Cluster A had higher values for VIs at A except for GNDVI_A.

Each of these two clusters was separated into two subclusters, A1 and A2 for landraces and B1 and B2 for modern cultivars. Subcluster A1 was represented mainly by south Mediterranean landraces (77%), including those from the east and west regions, whereas Subcluster A2 contained most of the north Mediterranean landraces (62%). East Mediterranean landraces were in a single cluster within A1, whereas west Mediterranean landraces were distributed in other clusters within A1. Differences among subclusters, A1 and A2 were due to higher NSm^2^ and TCARIOSAVI_PA in A1 and longer cycles until anthesis in A2, along with higher values for GA and VIs at PA. Regarding modern cultivars, Subcluster B1 was composed mainly of genotypes carrying the CIMMYT/ICARDA genetic background (SP6) (62%) and included the two landraces allocated to Cluster B mentioned above. Moreover, Subcluster B2 included 91% of the cultivars from SP4 (French and Italian modern cultivars). Most of the modern Balkan cultivars (SP5) were grouped in Subcluster B1. Subcluster B1 was characterized by higher TCARIOSAVI_PA and GA, whereas Subcluster B2 was characterized by higher NSm^2^, GNDVI_A and the rest of the VIs assessed in PA.

### Marker-Trait Associations

**Figure 4:**
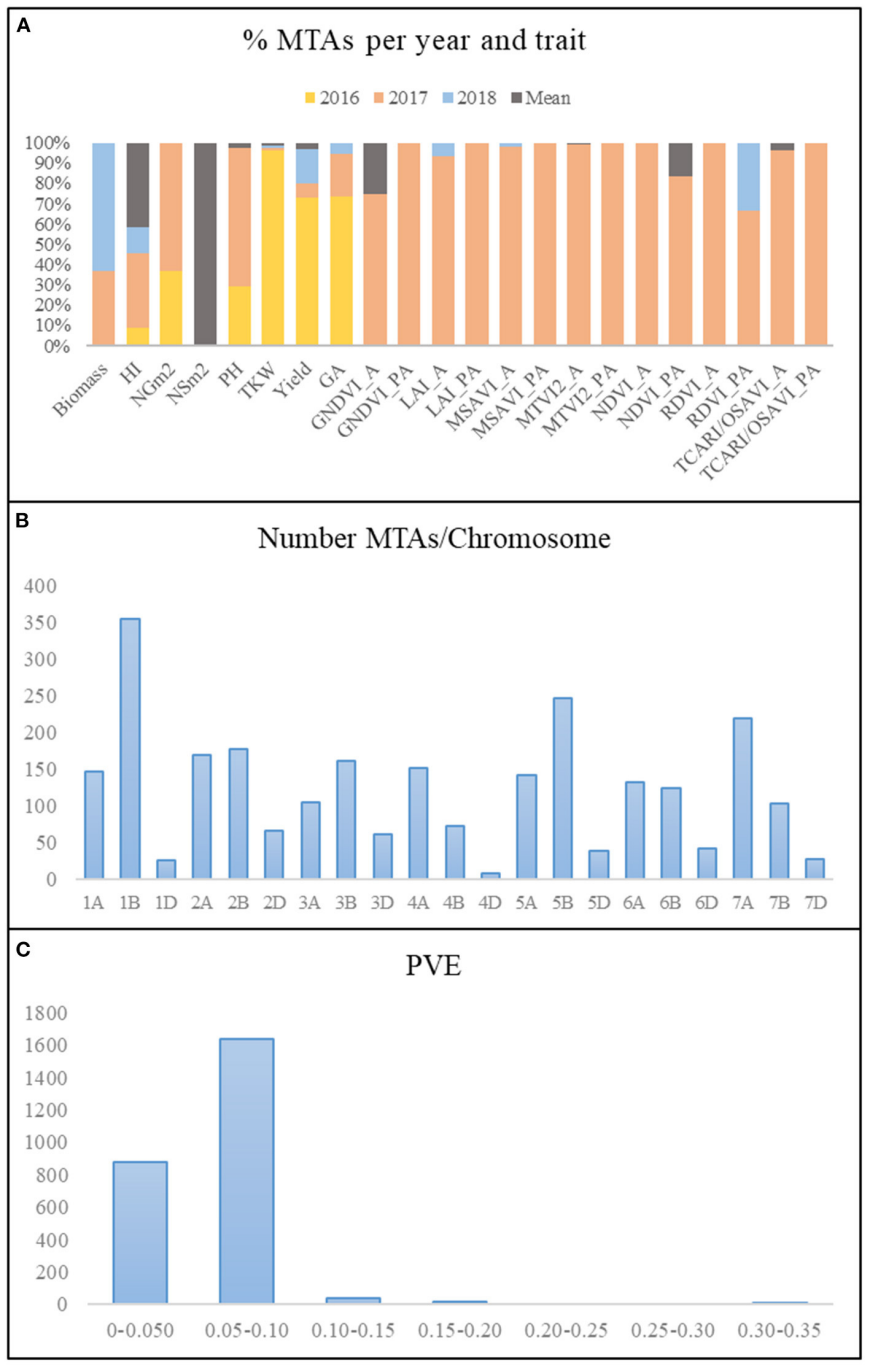
Summary of MTAs. **(A)** Percentage of MTAs per year and trait. **(B)** Number of MTAs per chromosome. **(C)** Phenotypic variance explained (PVE). MTAs, marker–trait associations; HI, harvest index; LAI, leaf area index estimated by MTVI2; NSm^2^, number of spikes per square meter; NGm^2^, number of grains per square meter; TKW, thousand kernel weight (g); PH, plant height; GA, green area from emergence until the first node; A, anthesis stage; PA, postanthesis.

A summary of the results of the genome-wide association study (GWAS) for all traits per year and for the mean values across years is reported in Figure 4. Due to the low number of marker trait associations (MTAs) showing significance above a false discovery rate (FDR) threshold at –log_10_ *P* > 4.8, a common threshold of –log_10_ *P* > 3, as reported in the literature (Wang et al., 2017, 2020; Condorelli et al., 2018; Mangini et al., 2018; Sukumaran et al., 2018), reported a total of 2,579 MTAs (Supplementary Table 4). Manhattan plots for each of the traits and year are represented in Supplementary Figure 1. The year 2017 presented the highest number of MTAs, 74% of the total number of MTAs, whereas 2018 and the mean across years presented the lowest number of MTAs, 3 and 4%, respectively (Figure 4A). During 2016, only MTAs related to agronomic traits were reported, accounting for 19% of the total MTAs across years, as no multispectral images were captured during that year.

The number of MTAs per chromosome for all years and for the mean values across years ranged from 9 on chromosome 4D to 354 on chromosome 1B (Figure 4B). Genome B accounted for 48% of the total MTAs, followed by genomes A and D with 41 and 11%, respectively. The percentage of MTAs with a phenotypic variance explained (PVE) lower than 0.10 was 97.5%, which agreed with the highly quantitative nature of the analyzed traits (Figure 4C).

A total of 815 MTAs were identified for seven agronomic traits (Supplementary Table 5). Yield showed the highest number of MTAs (368), most of them (268) from 2016, whereas only one association was found for NSm^2^ with the mean across years. MTAs for TKW were found mainly during 2016 (96%), and those for PH were found mainly during 2017 (68%).

A total of 1,764 MTAs over –log_10_ *P* > 3 were identified for 15 VI traits (Supplementary Table 5). Among them, 1,718 were detected a tor before A of green area (GA), and only 46 MTAs were identified at PA. Ninety-six percent of the MTAs were identified during 2017, which was the year characterized by the lowest rainfall. TCARIOSAVI_A was the trait with the highest number of MTAs (1,243), followed by MTVI2_A with 350.

To identify the genomic regions mostly involved in trait variation, QTL hotspots were identified using the QTL overview index defined by Chardon et al. (2004) for each cM of the genetic map reported by Wang et al. (2014). Confidence intervals (CIs) were calculated using the linkage disequilibrium (LD) decay for each chromosome reported by Rufo et al. (2019).

**Figure 5:**
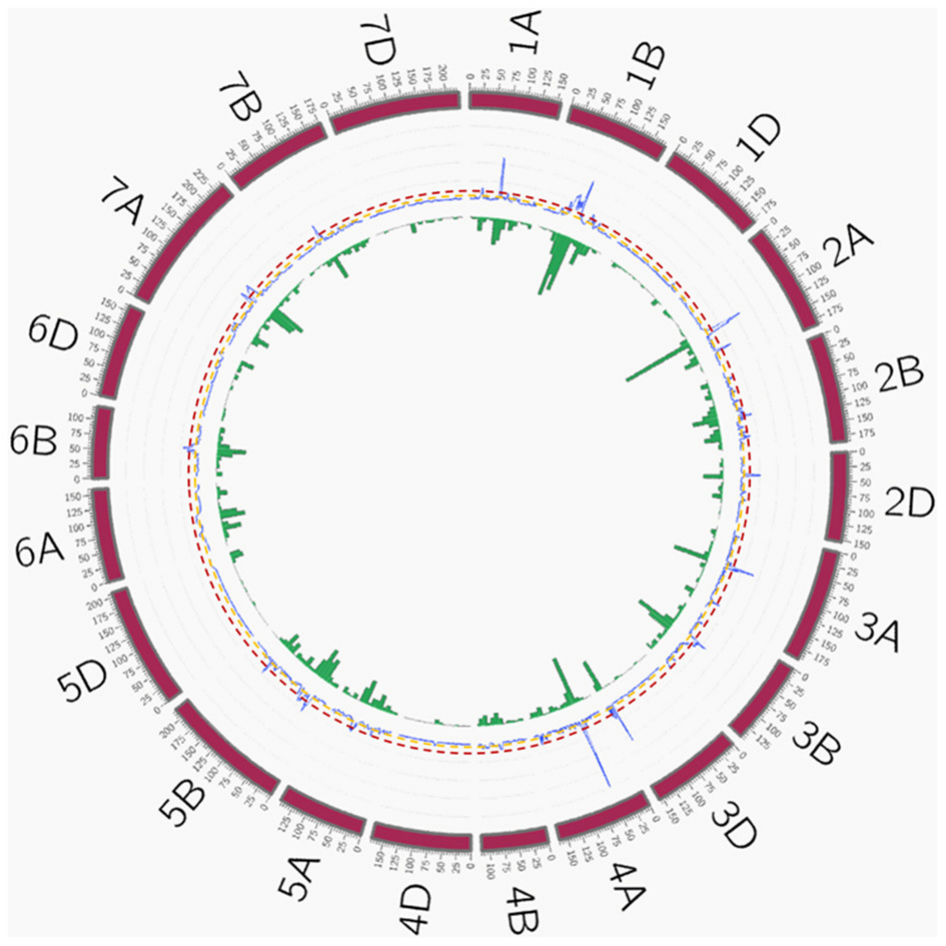
QTL overview index. The index values are represented along chromosomes as a blue line. Yellow and red dashed lines represent the thresholds for average (0.7) and higher values (3.5), respectively. Green bars below the QTL overview index represent the number of significant MTAs per 10 cM (–log_10_ *P* > 3). QTL, quantitative trait loci; MTAs, marker–trait associations.

A total of 209 peaks were identified using the mean of the overview index across the 21 chromosomes (0.7) as the threshold, whereas using a high threshold (3.5), a total of 41 peaks were detected (Figure 5). These 41 peaks were reduced to 28 QTL hotspots (Supplementary Table 6), 12 in genomes A and B and 4 in genome D. To simplify the search for candidate genes (CGs), quantitative trait loci (QTL) hotspots were excluded when (1) the centromere was included within the hotspot or the CI was higher than 35 Mb and (2) MTAs corresponded only to 1 year of field experiments. Eleven QTL hotspots grouping 295 MTAs remained for subsequent analysis (Table 8). As shown in Figure 5, hotspots defined by the QTL overview index correspond to genome regions with a higher number of MTAs.

**Table 8:** QTL hotspots identified for agronomic and remotely sensed VI-related traits.

**QTL hotspot**	**Position (cM)**	**MTAs**	**Max** **-log** ***P***	**Env**	**Number of** **traits**	**Left marker**	**Position (bp)**	**Right marker**	**Position** **(bp)**	**CI** **(Mb)**
QTL1A.1	24-29-31	18	18.4	2	2	BS00056550_51	7294564	Kukri_c29655_239	9579957	2.3
QTL1B.2	107-114-124	52	6.8	4	6	BS00072791_51	629262942	RFL_Contig2971_282	652455350	23.2
QTL2A.2	149-151-153	25	5.5	2	6	IAAV880	755788335	Tdurum_contig50839_593	758514679	2.7
QTL2D.1	39-41-45	29	6.2	2	6	Kukri_c16477_181	61979485	BS00067584_51	79416689	17.4
QTL3A.2	176-177-178	9	5.9	2	5	Excalibur_c77321_69	737299578	Tdurum_contig31235_99	739397160	2.1
QTL3D.1	142-143-144	32	7.0	2	5	Kukri_rep_c87658_1436	613696030	wsnp_Ex_c13629_21411429	609166802	4.5
QTL4A.2	150-151-153	8	5.3	2	5	Excalibur_c74390_108	733915685	RAC875_c11702_1015	739524596	5.6
QTL5B.2	54-61-64	51	6.2	3	6	BobWHIte_c47103_84	457342562	BS00039492_51	487602616	30.3
QTL5B.3	68-69-69	10	5.4	3	3	GENE-3574_643	519148851	TA002629-0202	526576640	7.4
QTL5B.4	159-161-163	21	18.2	3	8	wsnp_Ku_c3151_5892200	680852782	RAC875_c278_1801	683143220	2.3
QTL7A.1	110-114-119	40	6.5	2	4	BS00024786_51	79542753	Kukri_rep_c101532_1046	84767559	5.2
**QTL hotspot**						**Traits**				
QTL1A.1						GNDVI_A, TCARI/OSAVI_A				
QTL1B.2						Yield, HI, MTVI2_A, PH, TCARI/OSAVI_A, TKW				
QTL2A.2						Yield, HI, LAI_A, MTVI2_A, TCARI/OSAVI_A, TKW				
QTL2D.1						Yield, MSAVI_A, MTVI2_A, PH, RDVI_A, TCARI/OSAVI_A				
QTL3A.2						Yield, LAI_PA, MTVI2_A, PH, TCARI/OSAVI_A				
QTL3D.1						Yield, LAI_PA, MTVI2_A, PH, TCARI/OSAVI_A				
QTL4A.2						Yield, MSAVI_A, PH, RDVI_A, TCARI/OSAVI_A				
QTL5B.2						Yield, HI, MSAVI_A, RDVI_A, TCARI/OSAVI_A, TKW				
QTL5B.3						Yield, HI, TCARI/OSAVI_A				
QTL5B.4						Yield, HI, LAI_A, MSAVI_A, MTVI2_A, PH, RDVI_A, TCARI/OSAVI_A				
QTL7A.1						Yield, MTVI2_A, PH, TCARI/OSAVI_A				

*Positions are indicated in centimorgans (cMs) and base pairs (bp)*.

*MTAs, marker trait associations; Env, number of environments; CI, confidence interval; HI, harvest index; TKW, thousand kernel weight; LAI, leaf area index; PH, plant height; A, anthesis; and PA, postanthesis*.

### *In silico* Analysis of CGs

**Figure 6:**
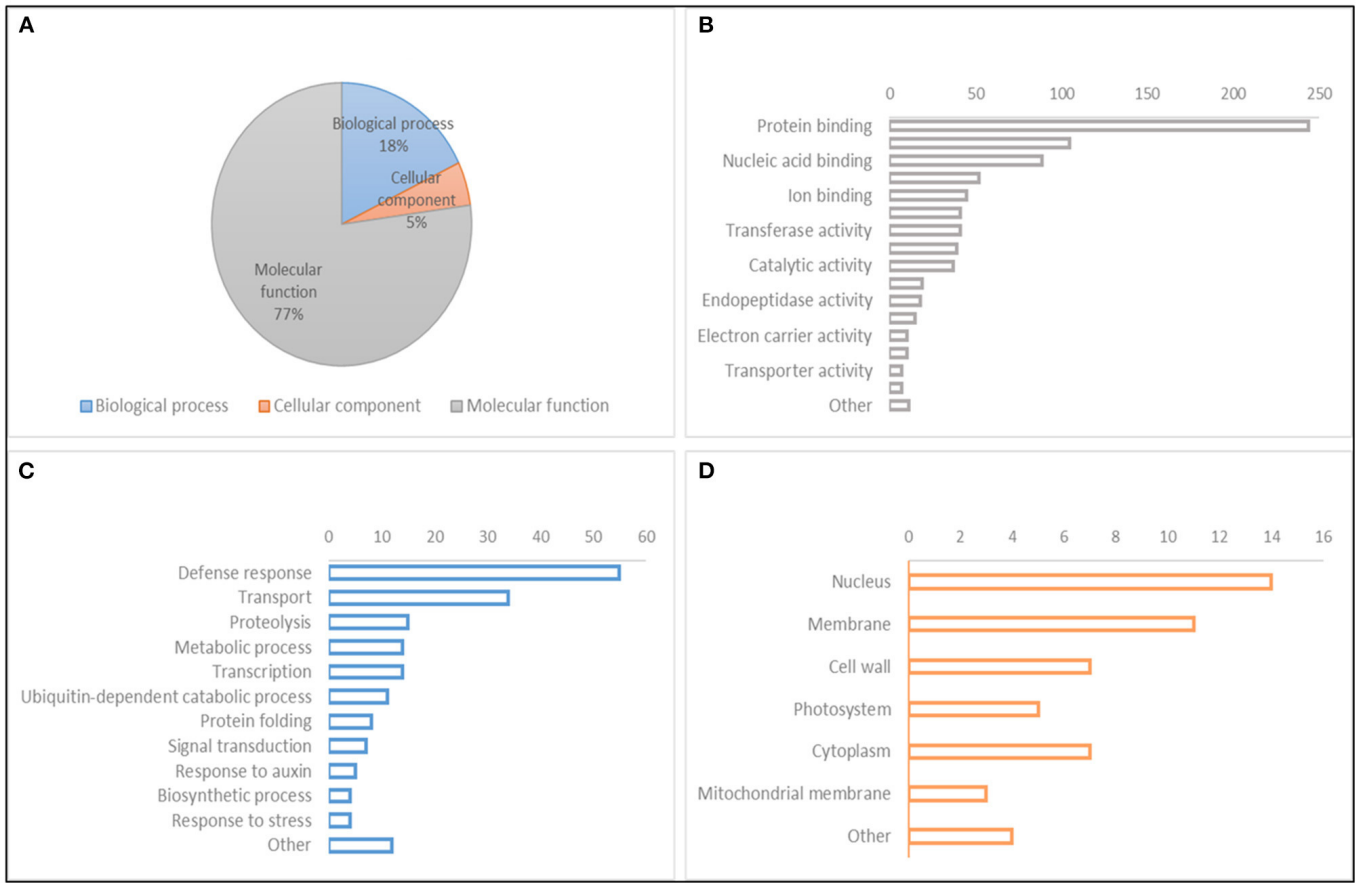
Gene Ontology (GO) classification of gene models within QTL hotspots. **(A)** GO hierarchy. **(B)** Molecular function. **(C)** Biological process. **(D)** Cellular component.

A search for CGs to study the relative gene expression levels under abiotic stress conditions and different tissues and developmental stages was performed within the QTL hotspot regions reported in Table 8 using the positions of flanking markers in the “Chinese Spring” reference genome (IWGSC, 2018) at https://wheat-urgi.versailles.inra.fr/Tools/JBrowse. A total of 1,342 gene models were detected, and to classify this information, Gene Ontology (GO) for 1,025 of the gene models (76%) was downloaded from https://wheat-urgi.versailles.inra.fr/Seq-Repository/Annotations (Figure 6; Supplementary Table 7). Seven hundred ninety-one CGs were classified according to molecular function (MF), 183 according to biological process (BP), and 51 according to cellular component (CC). The most represented CGs according to molecular function were “protein binding” (31%), “protein kinase activity” (13%), and “nucleic acid binding” (11%). According to BP, 30% of the CGs were involved in “defense response” and 19% in “transport.” Finally, according to CG, 27% of the product of CGs were in the nucleus, 22% in the membrane, and 14% in the cytoplasm and cell wall.

**Figure 7:**
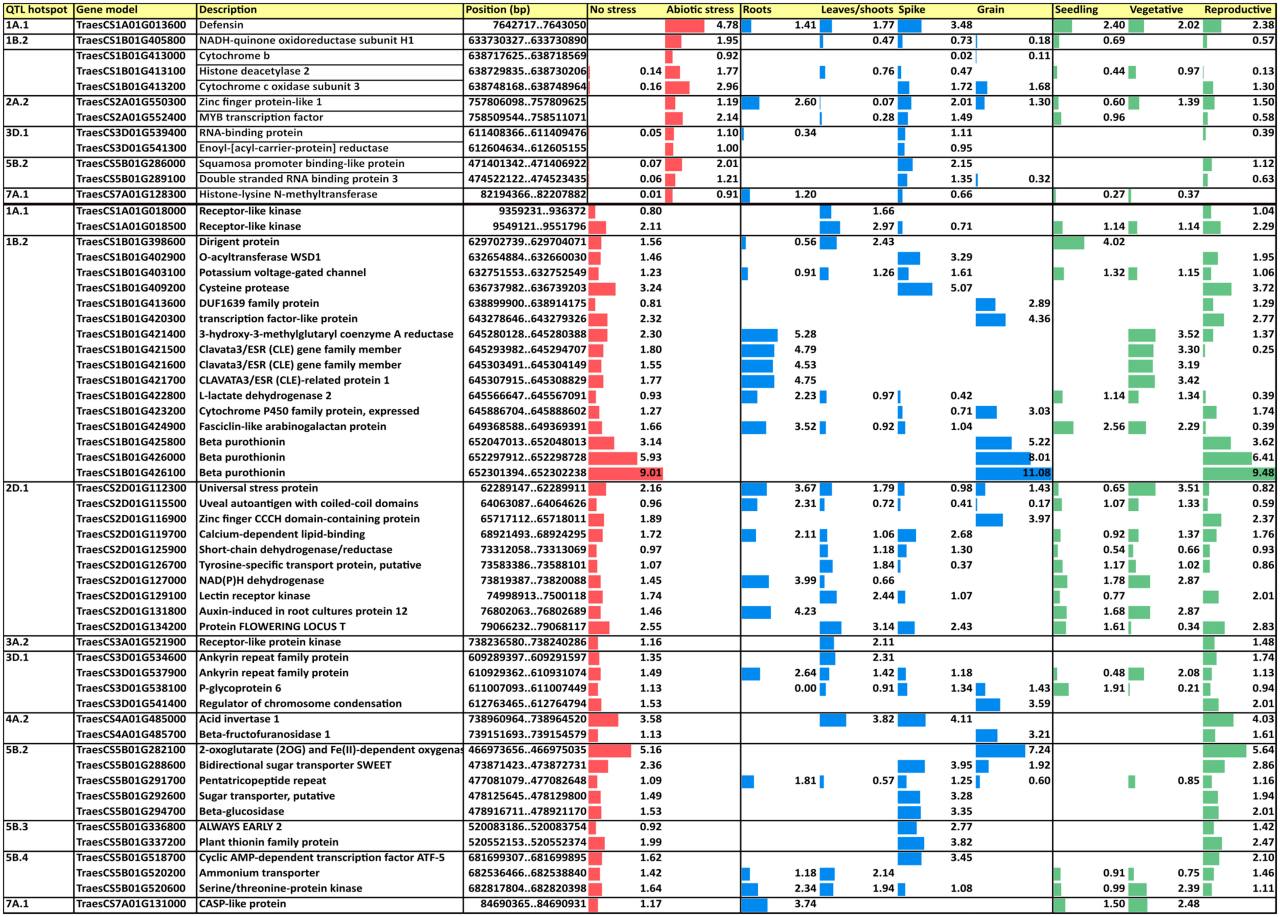
Upregulated and downregulated CGs under abiotic stress conditions in four tissues and three developmental phases. Values are based on log_2_ tpm. CGs, candidate genes; tpm, transcripts per million.

Subsequently, a search for differentially expressed genes (DEGs) under three abiotic stress conditions as reported in http://www.wheat-expression.com was carried out. These conditions included (1) drought and heat stress time-course in seedlings, (2) spikes with water stress, and (3) seedlings treated with polyethylene glycol (PEG) to simulate drought, and DEGs were analyzed in four tissues (roots, shoots/leaves, spikes, and grains) during different developmental phases (seedling, vegetative, and reproductive). A total of 12 CGs that were upregulated under abiotic stress were found in six QTL hotspots and 46 were found downregulated in 10 QTL hotspots (Figure 7).

Among the different upregulated DEGs, a defensin in hotspot QTL1A.1 showed the highest expression under abiotic stress conditions and was expressed in most of the tissues and all the developmental phases; it also showed the highest expression levels for each of the phases. All DEGs reported expression in the spikes with a range from 0.02 tpm for cytochrome b in QTL1B.2 to 3.48 tpm for defensin in QTL1A.1. Only four DEGs were expressed in the roots and five in the leaves/shoots and grain. Only zinc finger protein-like 1 in QTL2A.2 was expressed in all four plant tissues, showing the highest expression in roots. Regarding the developmental phase, no expression was reported in any stage for two DEGs, cytochrome b in QTL1B.2 and enoyl-[acyl-carrier-protein] reductase in QTL3D.1. The reproductive phase had the highest number of DEGs (nine out of 10 showing expression), whereas 6 were expressed in the seedlings and only four were expressed during the vegetative phase.

Among the downregulated DEGs, the hotspot QTL1B.2 showed the highest number of downregulated DEGS (16), whereas QTL2A.2 did not show any of them. Three DEGs showed expression in all tissues, whereas any of them were downregulated in all of tissues. About six DEGs were expressed only in roots under non-stressed conditions, three in leaves/shoots, seven in spikes, and nine in grains, whereas DEGs non-expressed under abiotic stress in only one tissue corresponded to one in roots and six in grains. According to the developmental phase, 12 DEGs were expressed in all of them, whereas any DEG was downregulated in all of them. One DEG was expressed only in the seedling stage at normal conditions, whereas two were expressed only in the vegetative and 22 in the reproductive stages.

## Discussion

The current study was conducted under typical Mediterranean environmental conditions, with a pattern of increasing temperatures during the spring and an irregular distribution of rainfall across years. A genome-wide association study (GWAS) panel of 354 bread wheat genotypes, including Mediterranean landraces and modern cultivars, was grown for 3 years under these conditions in northeastern Spain. Given that the decrease in the genetic diversity of wheat occurred during the second half of the twentieth century, associated with the introduction of high-yielding semidwarf cultivars (Autrique et al., 1996), landraces are considered a natural reservoir of genetic variation within the species and an invaluable source of new alleles to widen the genetic variability in breeding populations, particularly for traits regulating adaptation to suboptimal environments (Lopes et al., 2015). Recent studies have demonstrated the scarce use of wheat landraces in breeding programs in the past, as suggested by the high genetic diversity and defined population structure among landrace and modern cultivar subpopulations (Soriano et al., 2016; Rufo et al., 2019).

### Phenotypic Performance

The high heritability reported for the agronomic traits, reaching 0.9 for yield and 0.8 for HI, NGm^2^ and PH indicated that genetic differentiation among landraces and modern cultivars played a predominant role in determining the variation for these traits.

The ANOVAs showed a large effect of subpopulation (SP) on the phenotypic expression of the agronomic traits, whereas year showed the largest effect for most of the VIs, followed by the year × SP interaction, with the SP effect being the lowest. The variability in agronomic traits was mostly caused by the different agronomic performances of wheat landraces and modern cultivars, as reported in previous studies (Soriano et al., 2016; Royo et al., 2020). On the other hand, the high year effect on VIs was likely due to the contrasting water availabilities during the 2 years in which images were acquired by unmanned aerial vehicles (UAVs) in the experimental fields. This was not an unexpected result given that the decrease in the rate of growth of wheat caused by drought stress results in a severe reduction in total aboveground biomass (Royo et al., 2004; Casadesús and Villegas, 2014).

Yearly variation in weather conditions, particularly water input, resulted in a yield range from 7.4 t/ha in 2017, the driest year, to 7.9 and 8.0 t/ha during the years with higher rainfall. Even with the low water input, the average experimental yields were higher than expected in a severe drought environment. The number of spikes and grains per unit area were the highest in 2018, the wettest year, but were the lowest in 2017. Grain weight showed the opposite pattern, suggesting that under drier and hotter conditions, cultivars filled their grains at a higher rate (1.29 g/day in 2017 and 1.06 g/day in 2016 and 2018), thus showing a shorter grain filling duration (GFD) in 2017. The high yields recorded, considering the rainfed conditions of the field trials, could be attributed to the high soil fertility (~3% of organic matter) and the superficial subsoil water layer at this site (Royo et al., 2021).

From a genetic viewpoint, a clear separation was observed between landraces and modern cultivars for most of the agronomic traits, which can be attributed to the improvement achieved by breeding. As expected, the yield was negatively correlated to plant height (PH) as reported previously by Royo et al. (2020). Among landraces, those from northern Mediterranean countries characterized by high rainfall and lower temperatures (Royo et al., 2014) showed higher yields due to an increase in the number of grains per unit area and grain weight. These genotypes showed longer cycles until A and a shorter grain filling duration, although this last trait was not statistically significant. Landraces from the eastern Mediterranean countries showed lower yields, a lower number of grains, and lighter grains but an increase in the number of spikes per unit area compared with landraces from the northern Mediterranean countries. Similar results for the east Mediterranean landraces were previously reported in durum wheat by Soriano et al. (2018) and Roselló et al. (2019b), suggesting an adaptation of landraces from this area to warmer environments, which has been associated with the allelic constitution of vernalization and photoperiod genes (Royo et al., 2020). The results of the current study are in agreement with the previous research reporting a tendency for wheat to increase the number of ear-bearing tillers as an adaptation strategy under heat stress (Hütsch et al., 2019) and to increase the number of spikes per unit area in genotypes adapted to dry and warm areas compared to genotypes adapted to wetter and colder areas (Royo et al., 2014, 2020). Among modern cultivars, significant differences were mainly found between SP4 (cultivars from France and Italy) and the other two SPs (Balkans and CIMMYT-ICARDA-derived germplasm). These results suggest that breeding in France and Italy was in the direction of increasing yield by increasing the number of spikes and grains per unit area, whereas the other SPs showed higher thousand kernel weight (TKW). In addition, the regression results of the modern set suggested a high impact of genetic population structure on the number of grains per unit area. Cultivars derived from CIMMYT and ICARDA germplasms reached A earlier, up to 8 days earlier compared with Balkan cultivars and 6 days earlier compared to French and Italian cultivars, which was in line with the high *R*^2^ values obtained in the relation between the modern set structure and GS65. This earliness can help these cultivars from warmer regions avoid heat stress at the end of flowering.

All traits related to high-throughput phenotyping (HTP) showed significant differences between years before and after anthesis (A), showing higher values for most of the VIs in 2018 than in the previous year. These higher values agree with the rainfall recorded for both years, which was significantly lower in 2017 than in 2018, mostly during the grain filling period. Furthermore, the difference in the mean values between growth stages was much higher in 2017. This result could be explained by the water scarcity particularly affecting the PA stage, which results in an important loss of chlorophyll content during the grain filling period; therefore, VIs using bands mostly placed in the near-infrared (NIR) and green regions showed lower values (Adamsen et al., 1999). It was supported by the high and positive correlations values between GA and GNDVI and LAI at post A stage, which was the case in 2018. Even though water stress affects the growth of wheat, the effects are higher during the grain filling duration (GFD) (Moragues et al., 2006). Thus, the leaf area index (LAI) and green normalized difference vegetation index (GNDVI) values decreased at the end of the growing cycle due to a low chlorophyll content associated with senescence during the grain filling period (Rufo et al., 2021). In addition, Gitelson et al. (2002) reported that the sensitivity of the green band was higher than that of the red band when the vegetation fraction was more than 60%, so vegetation indices using green wavelengths perform better at high LAI values, which in wheat under Mediterranean conditions are the highest at booting (Aparicio et al., 2000; Royo et al., 2004; Kyratzis et al., 2017; Rufo et al., 2021). This agreed with the high and positive correlation values between GS65 and GNDVI and LAI, indicating that more days until A provides a high green LAI at postanthesis (PA) stage in wet years as 2018. TCARI/OSAVI had higher PA for both years. This agreed with the results of Zarco-Tejada et al. (2005), who reported that in advanced growth stages, chlorophyll indices, such as TCARI performed better due to being less sensitive to the loss of turgor and leaf drop. In fact, these authors also stated that the different patterns of the indices across growth stages suggested that chlorophyll-related indices are more suitable closer to harvest, while structural indices related to canopy light scattering and growth are better for early stages.

Differences in the mean values of SPs were found in the two growth stages, with the highest values mainly found at A based on the differences among years, thus highlighting the effect of PA senescence on the chlorophyll content. Landraces and modern cultivars showed significant differences in the LAI and structural vegetation indices (VIs) at A, and these values were higher in the landraces. As reported in previous studies in durum wheat (García Del Moral et al., 2005; Soriano et al., 2018), landraces are characterized by their tolerance to water scarcity and their superior water use efficiency before A compared to modern cultivars (Subira et al., 2015). The SPs showing the highest mean values for the LAI and VIs at PA were those including landraces from the north of the Mediterranean basin (SP2) and modern cultivars from France and Italy (SP4). Landraces from SP2 are better adapted to colder and wetter environments than landraces originating in the southern part of the Mediterranean basin. This adaptation pattern has been associated with the greatest early soil coverage and more aboveground biomass along the whole cycle length (Royo et al., 2014, 2021). For this reason, the canopy remains green much longer in landraces from the northern Mediterranean countries than in those from the southern Mediterranean countries (Royo et al., 2014). The same pattern was found in modern cultivars, with GNDVI values remaining higher than those of landraces after A and being significantly different among modern subpopulations. These results agreed with those from the relationship between structure and GNDVI_PA, where the modern set showed the highest *R*^2^ values according to the differences found in GNDVI mean values among modern SPs. These results and the capacity to discern between landrace and modern SPs regarding the VI values at A proved the accuracy of HTP in characterizing populations. Several studies have stated the potential of remote sensing for assessing agronomic traits by screening hundreds of plots in a short period of time, minimizing replications (Araus et al., 2018; Gracia-Romero et al., 2019; Juliana et al., 2019). Furthermore, various authors have stressed the suitability of using VIs measured early in the season for grain yield forecasting (Aparicio et al., 2000).

Bidimensional clustering was helpful to jointly visualize the results obtained by Tukey's tests. Moreover, clustering of agronomic and HTP data revealed similarity with the separation obtained by Rufo et al. (2019) using SNP markers and SPs defined based on the structured collection. In both the cases, a clear differentiation among landraces and modern cultivars was observed, which resulted in separation into two main clusters. Within the landrace cluster, A separation was observed between landraces from the northern and southern Mediterranean countries, thus including landraces from SP2 in one cluster and those from SP1 and SP3 in the other cluster, with different groupings among them. Modern cultivars of SP6 (CIMMYT-ICARDA) clustered separately from the French and Italian cultivars (SP4), whereas modern cultivars from the Balkans grouped mostly with SP6. Although these two SPs were separated genetically, no significant differences were found for the agronomic traits, except for phenology, and regarding the VIs, no differences were found at A. Two landraces (TRI 11548 and 1170) were included within modern cultivars from CIMMYT-ICARDA and the Balkans. These two landraces were characterized by a longer GFD, higher HI, and lower number of spikes per unit area than the average for landraces. Landrace TRI 11548 from Iraq also showed higher yield and grain weight than other landraces, so it probably resulted from a selection made in an early landrace population.

### Marker Trait Associations

Dissecting the genetic basis of complex traits in plant breeding is essential to tackle molecular-based approaches for crop improvement. Several efforts have been previously made to identify quantitative trait loci (QTLs) and marker–trait associations (MTAs) associated with traits of interest to carry out marker-assisted selection (MAS) approaches and the introgression of alleles of commercial interest in adapted phenotypes.

The highest number of MTAs related to agronomic traits was found in 2016, while 96% of MTAs related to VIs, GA, and the LAI were identified in 2017. It has been reported that under contrasting conditions, the G × E interaction could affect the identification of stable associations among different environments (Mwadzingeni et al., 2017), which could explain the difference in the number of significant associations among the 3 years of field trials. The highest number of associations for yield and TKW in 2016 could be due to the moderate amount of water input (rainfall) during the spring, together with the longest grain filling duration, as reported in previous studies where grain weight predominantly enhanced yield in wet environments (García Del Moral et al., 2003; Moragues et al., 2006; Royo et al., 2006). Moreover, Royo et al. (2020) found that genotypes with longer GFDs could have greater opportunities to increase grain weight in favorable growing seasons than in warmer and drier seasons. The elevated number of VI-related MTAs found in the driest year (2017) could be explained by the higher variability in traits related to leaf biochemical properties or canopy structural attributes within the set of genotypes grown in environments with water scarcity (Rufo et al., 2021). The highest number of MTAs was identified for PH in 2017, when the coefficients of variation (CV) was higher for this trait. Qaseem et al. (2019) suggested that taller genotypes under drought stress could increase yield accumulation and convert more assimilates into grain. Of the 1,764 MTAs detected for VIs, 1,718 were found at A, with 1,243 for TCARI/OSAVI. This result could be explained by the significant differences found between landraces and modern SPs at A for traits related to HTP. The highest variability was found when comparing SP4 with the rest of the SPs for TCARI/OSAVI, which could explain the elevated number of MTAs for this trait. The distribution of the MTAs across genomes agreed with the results of Rufo et al. (2020), with a similar number in the A and B genomes (41 and 48%, respectively) and the remaining 11% in the D genome. These results are consistent with those of previous studies (Chao et al., 2010; Wang et al., 2014; Gao et al., 2015), which attributed these values to the lower genetic diversity and higher LD found in the D genome of bread wheat compared with genomes A and B (Rufo et al., 2019).

### QTL Hotspots

To reduce the complexity of the high number of identified MTAs, QTL hotspots were defined using the QTL overview index proposed by Chardon et al. (2004). Although this statistic was initially used for classical biparental QTL analysis, we adapted it to GWAS using the confidence intervals (CIs) of the MTAs as the distance of LD decay for each of the chromosomes. As reported in Figure 5, QTL hotspots defined by the high-value threshold of the overview index corresponded to genome regions with a higher MTA density, thus supporting the suitability of this approach in GWAS. To identify genome regions previously mapped in locations similar to our QTL hotspots and to detect new loci controlling agronomic traits and VIs, a comparison with previous GWAS studies and/or meta-QTL analysis reporting yield- and VI-related traits was conducted. Seven of the 11 QTL hotspots have been described previously in the literature. When compared with the meta-QTL analysis reported by Liu et al. (2020) in bread wheat, the QTL hotspots QTL1B.2 and QTL2D.1 were located at similar positions as MQTL1B.7 and MQTL1B.8 and MQTL2D.3 and MQTL2D.4, respectively, controlling grain yield, grain number, and TKW under drought and heat stress. QTL1B.2 was also in the homologous region of QTL IWB50693 in durum wheat controlling spike length (Anuarbek et al., 2020), QSN.caas-1BL controlling NSm^2^ identified by Gao et al. (2015) in bread wheat and IWB3330 controlling the normalized chlorophyll pigment ratio index (NCPI) identified by Gizaw et al. (2018) in bread wheat. Gao et al. (2015) also found three QTLs for TKW, chlorophyll content, and NDVI located in a common region with the hotspot QTL5B.2. This QTL hotspot was also detected in a similar region as QTL yield/root_5B.1 controlling grain yield and shoot length identified by Rufo et al. (2020). QTL1B.2 and QTL5B.2 were found to have homology with several studies. This was an expected result, since they were the longest hotspots including the highest number of MTAs. The genomic regions for QTL hotspots QTL5B.4 and QTL7A.1 were also found in common with three QTLs identified by Anuarbek et al. (2020) controlling the number of fertile spikes and TKW in durum wheat under rainfed conditions. Hotspots QTL1A.1 and QTL2A.2 shared a common position with mtaq-1A.2 reported by Roselló et al. (2019a) in durum wheat and QTL yield/root_2A2 identified by Rufo et al. (2020) in bread wheat, respectively, controlling root-related traits and grain yield in bread wheat.

The detection of these regions in common with other studies opens the opportunity to a pyramid of different QTLs with pleiotropic effects in future breeding approaches. Moreover, the use of the reference genome sequence makes it possible to rapidly identify common molecular markers to be used in MAS.

### Candidate Genes

Gene annotation from the “Chinese spring” reference genome sequence (IWGSC, 2018) allowed us to identify 1,342 gene models within the 11 QTL hotspots. Candidate gene (CG) mining was performed by searching for differentially expressed genes (DEGs) upregulated and downregulated under drought conditions in different tissues and developmental stages through *in silico* analysis at http://www.wheat-expression.com.

Four CGs that were upregulated under drought stress have been previously reported in the literature to be involved in stress resistance. Among them, in QTL hotspot 1A.1, a defensin protein (TraesCS1A01G013600) was found to show the highest expression under drought stress. According to Kumar et al. (2019), although defensins are mainly involved in antifungal responses, the defensin gene *Ca-AFP* from chickpea in transgenic *Arabidopsis* plants was overexpressed under drought stress and induced a higher germination rate, root length, and plant biomass. Two gene models enhancing drought and heat stress tolerance were found in QTL hotspot 2A.2: the gene model TraesCS2A01G550300 encoding a zinc finger protein, as reported by (Yoon et al., 2014) in poplar, and the gene model TraesCS2A01G552400 encoding a MYB transcription factor, which was described by Zhao et al. (2018). *TaMYB31* from wheat is transcriptionally induced by drought stress in transgenic *Arabidopsis* plants (Zhao et al., 2018). Finally, in QTL hotspot 5B.2, a squamosa-binding protein was identified (TraesCS5B01G286000). These protein families have been found to be involved in several biological processes. Cao et al. (2019), in expression studies of the Squamosa binding protein from wheat *TaSPL16*, found that this gene was highly expressed in young panicles but expressed at low levels in seeds, in agreement with the expression profile of TraesCS5B01G286000 found in our study. The ectopic expression of *TaSPL16* in *Arabidopsis* produced a delay in the emergence of vegetative leaves and early flowering and affected yield-related traits. Other gene models upregulated under drought stress, as reported in the RNA-seq analysis from Ramírez-González et al. (2018), such as NADH-quinone oxido reductase, cytochrome b, histone deacetylase 2, RNA-binding protein, enoyl-[acyl-carrier-protein] reductase, double-stranded RNA binding protein 3, and histone-lysine N-methyltransferase, have not been related to drought stress tolerance in the literature, and further experiments are required to assess their expression under drought stress conditions. On the other side, 46 gene models were shown to be downregulated under drought stress. However, the decrease in the expression level seems to be more associated with the breakdown of the physiological functions due to drought than a causal effect in response to the stress.

## Conclusions

The use of local landraces in breeding programs is considered a valuable approach to broadening the genetic variability of crops lost during the breeding process and improving traits of commercial importance. The results reported in the present study evidenced the selection for grain yield, HI, NG m^2^, and GNDVI_PA during the breeding process.Whereas differentiation among landraces were found for agronomic and VIs (grain yield, TKW, GNDVI_PA, and GA), in modern cultivars SPs differentiation were mainly due to GS65, GNDVI, and NDVI_PA. The use of a statistical approach as the QTL overview index for the definition of QTL hotspots resulted successful for the identification of consensus genome regions including most of the stable marker trait associations across years. The results of this study will be useful for our wheat breeding program by the selection of the appropriate genotypes carrying favorable alleles for the differential traits that will be useful for designing new crosses.

Using *in silico* approaches allowed gene mining in QTL hotspots, thus facilitating CG identification.

## Data Availability Statement

The original contributions presented in the study are included in the article/Supplementary Material, further inquiries can be directed to the corresponding author/s.

## Author Contributions

JS: conceptualization and supervision. RR, AL, ML, JB, and JS: methodology. RR, JB, and JS: formal analysis. RR, AL, and JS: data curation. RR: writing—original draft preparation. RR, ML, JB, and JS: writing—review and editing. ML and JS: project administration and funding acquisition. All authors have read and agreed to the published version of the manuscript.

## Funding

This study was funded by the projects AGL2015-65351-R and PID2019-109089RB-C31 from the Spanish Ministry of Science and Innovation.

## Conflict of Interest

The authors declare that the research was conducted in the absence of any commercial or financial relationships that could be construed as a potential conflict of interest.

## Publisher's Note

All claims expressed in this article are solely those of the authors and do not necessarily represent those of their affiliated organizations, or those of the publisher, the editors and the reviewers. Any product that may be evaluated in this article, or claim that may be made by its manufacturer, is not guaranteed or endorsed by the publisher.

## Supplementary Material

Supplementary Figure 1 Manhattan plots for the analyzed traits during the 3 years and the mean across years.

Supplementary Table 1 Genotypes used in this study.

Supplementary Table 2 Phenotypic values of the analyzed traits for the 3 years of field trials.

Supplementary Table 3 Boxplots and summary statistics for the analyzed traits. Coefficients of variation for agronomic traits among the 3 years are included.

Supplementary Table 4 Total number of marker–trait associations (MTAs) per year and trait with –log_10_ *P* > 3.

Supplementary Table 5 Marker trait associations (MTAs).

Supplementary Table 6 QTL hotspots.

Supplementary Table 7 Gene models within QTL hotspots.
