# Supplementary material for: Identification of Quantitative Trait Loci Hotspots Affecting Agronomic Traits and High-Throughput Vegetation Indices in Rainfed Wheat
Source: Front Plant Sci. 2021 Sep 20;12:735192. doi: 10.3389/fpls.2021.735192 (PMC8489662; doi:10.3389/fpls.2021.735192)
Supplement: Supplementary Table 4 — Total number of marker–trait associations (MTAs) per year and trait with –log10 P > 3. [file Table_4.docx]

Supplementary Table S4. Total number of MTAs per year and trait with -log_10_*P* > 3.

| Trait | 2016 | 2017 | 2018 | Mean | Total |
| --- | --- | --- | --- | --- | --- |
| Biomass | 0 | 7 | 12 | 0 | 19 |
| HI | 4 | 17 | 6 | 19 | 46 |
| NGm^2^ | 11 | 19 | 0 | 0 | 30 |
| NSm^2^ | 0 | 0 | 0 | 1 | 1 |
| PH | 65 | 150 | 0 | 6 | 221 |
| TKW | 125 | 2 | 1 | 2 | 130 |
| Yield | 268 | 26 | 62 | 12 | 368 |
| GA | 14 | 4 | 1 | 0 | 19 |
| GNDVI_A | - | 3 | 0 | 1 | 4 |
| GNDVI_PA | - | 8 | 0 | 0 | 8 |
| LAI_EST_A | - | 14 | 1 | 0 | 15 |
| LAI_EST_PA | - | 9 | 0 | 0 | 9 |
| MSAVI_A | - | 50 | 1 | 0 | 51 |
| MSAVI_PA | - | 3 | 0 | 0 | 3 |
| MTVI2_A | - | 348 | 0 | 2 | 350 |
| MTVI2_PA | - | 5 | 0 | 0 | 5 |
| NDVI_A | - | 1 | 0 | 0 | 1 |
| NDVI_PA | - | 5 | 0 | 1 | 6 |
| RDVI_A | - | 35 | 0 | 0 | 35 |
| RDVI_PA | - | 4 | 2 | 0 | 6 |
| TCARIOSAVI_A | - | 1195 | 1 | 47 | 1243 |
| TCARIOSAVI_PA | - | 9 | 0 | 0 | 9 |

GS65, number of days from sowing to anthesis; GFD, grain filling duration; HI, harvest index; NSm2, number of spikes per square metre; NGm2, number of grains per square metre; TKW, thousand kernel weight; LAI, leaf area index; PH, plant height; GA, green area; A, anthesis; PA, post anthesis.
